# Monitoring heavy metals, residual agricultural chemicals and sulfites in traditional herbal decoctions

**DOI:** 10.1186/s12906-017-1646-y

**Published:** 2017-03-14

**Authors:** In-Sil Yu, Jeong-Sook Lee, Sung-Dan Kim, Yun-Hee Kim, Hae-Won Park, Hoe-Jin Ryu, Jib-Ho Lee, Jeong-Mi Lee, Kweon Jung, Cheol Na, Jin-Yong Joung, Chang-Gue Son

**Affiliations:** 1 Kangbuk Agro-Fishery Products Inspection Center, Seoul Metropolitan Government Research Institute of Public Health and Environment, # 5 Yangnyeongjungang-ro, Dongdaemun-gu, Seoul, 130-864 Republic of Korea; 2 Seoul Korean Medical Association, # 420 Kosanja-ro, Dongdaemun-gu, Seoul, 130-817 Republic of Korea; 3 Liver and Immunology Research Center, Daejeon Oriental Hospital of Daejeon University, # 176-9 Daeheung-ro, Jung-gu, Daejeon, 302-724 Republic of Korea

**Keywords:** Herbal decoction, Quality control, Contamination, Heavy metals, Pesticides, Sulfur dioxide

## Abstract

**Background:**

Asian traditional herbal preparations are frequently considered for the contamination with undeclared toxic or hazardous substances. The aim of this study was to determine the toxic heavy metals, pesticides and sulfur dioxide in decoctions that is a common form of final utilization in Korea.

**Methods:**

A total of 155 decoctions composed of multi-ingredient traditional herbs were randomly sampled from Seoul in Korea between 2013 and 2014. For each decoction, the concentrations of four heavy metals (arsenic, cadmium, lead and mercury), 33 pesticides and sulfur dioxide were analyzed using inductively coupled plasma mass spectrometry (ICP-MS), mercury analyzer, gas chromatography/nitrogen phosphorous detector (GC/NPD), gas chromatography/micro electron capture detector (GC/μECD), and Monier-Williams method respectively.

**Results:**

One hundred fifty-two of One hundred fifty-five decoctions (98.1%) contained one of three heavy metals (96.1% for As, 97.4% for Cd, and 90.3% for Pb, 0.0% for Hg). Their average concentrations (77.0 ± 79.7 ug/kg for As, 20.4 ± 23.7 ug/kg for Cd, and 68.8 ± 76.5 ug/kg for Pb) were approximately 20% of the maximum allowable limits of vegetable or ginseng beverage described in the Korean Food Standard Codex while their 95th percentile concentrations were below than the guideline for them. None of 33 pesticides was detected in 155 decoction samples, and only one sample showed over limit of detection for residual sulfites.

**Conclusions:**

This study support that the contained status of toxic heavy metals, pesticides and sulfur dioxide in herbal decoctions are currently within safe level in Korea, and provide a reference data for the further studies focused on the safety herbal preparations.

**Electronic supplementary material:**

The online version of this article (doi:10.1186/s12906-017-1646-y) contains supplementary material, which is available to authorized users.

## Background

Herbal medicines have been used to manage various diseases and ailments for thousands of years particularly in East Asia, and herbal products became popular worldwide. The global market for herbal products is continuously growing, and reached US $83 billion in 2012 [1]. About 40% of individuals in Korea and China, and 18% of adults in the United States adopt herbal remedies to treat illnesses [2–4]. However, with the ever-increasing use of herbal medicines worldwide, many concerns have been raised regarding especially the safety and quality control of medicinal plant materials and herbal products [5, 6].

Safety and quality of herbal medicines are affected by many factors, intrinsic factor like species differences and extrinsic factors including environment, collection methods, cultivation, harvest, post-harvest processing, transport, and storage practices [7]. Quality control directly impacts not only the safety of herbal medicinal products also their efficacies [8]. Compared with synthetic drugs, assurance of quality control of herbal drugs, determining identity, purity, content, and biological property, are much more complex [9]. In addition, adulteration with undeclared other substances and contamination with undeclared toxic or hazardous substances are most likely to be found in herbal materials or herbal products [10]. The toxic heavy metals, residual pesticides or improper use of sulfites are regarded as potent risk factors in use of herbal medicines because they can be easily contaminated in herbal materials due to soil pollution and process of cultivation, harvesting and storage [11–13].

Those contaminants are known to be harmful in human health under certain levels, and then the allowable limits in medicinal herbs are under strict regulation by various countries including Korea [14]. The medicinal herbs are generally utilized as decoction forms of multiple herbal formulas especially in East Asia countries, which can change the quantities of contaminants in process of decoction [15]. In contrast many studies for quantification of those contaminants in herbal materials, very few information have been conducted for the herbal decoction to date.

This study firstly presents the levels of four toxic heavy metals, 33 pesticides and sulfites in 155 decoctions collected randomly from Seoul in Korea.

## Methods

### Collection of decoctions

One hundred fifty five decoctions (155 different formulae) were randomly collected from 51 oriental clinics (89 formulae), 7 herbal pharmacies (31 formulae) and 10 herbal medicine shops (35 formulae) between September 24 2013 and May 4, 2014 (Additional file 1: Table S1). The decoctions are generally prepared by adding 200 g of medicinal herbs to 1 L of water and boiling for 2 h. Each decoction contained approximately 100 ml of herbal solution in plastic bag. This one bag of decoction corresponds to a dose for an adult patient, which an adult generally takes 200 ml (two bags) as daily clinical dose.

### Analyses of heavy metals

#### Sample digestion and determination of element concentrations

Three heavy metals including arsenic (As), cadmium (Cd) and lead (Pb) were analyzed in decoction samples a using Inductively Coupled Plasma Mass Spectrometry (ICP-MS) (Agilent 7500ce, Agilent, Tokyo, Japan). Multi-element Calibration Standard 2A (Agilent, Santa Clara, CA, USA) containing 100 mg/L of Pb, Cd and As were used for external calibration. Working standards were prepared daily in 5% of HNO_3_ (70%, v/v, Dong Woo Fine-Chem Co., Iksan, Korea). Eight standards were prepared at concentrations ranging from 0 to 100 ug/L.

Samples were digested using a microwave digestion system (MARS 5; CEM, Matthews, NC, USA). Before use, the sample vessels were decontaminated in a bath of 10% of nitric acid, then rinsed with ultrapure water (18.2mΩ) (MQ gradient; Millipore, Bedford, MA, USA) and dried in a 40 °C oven. 2 g of decoction samples were weighed precisely in digestion vessels and wet-oxidized with 12 mL ultrapure HNO_3_ in the microwave digestion system. One randomly selected vessel was filled with reagents only and taken through the entire procedure as a blank. The microwave digestion system was sealed up and heated for over 15 min at 190 °C by applying 1200 W, and the sample was digested for 15 min. After cooling at room temperature, sample solutions were quantitatively transferred into 50 mL polyethylene flasks. The digested samples were then filled with ultrapure water to the final volume before analysis by ICP-MS. The operation parameters of ICP-MS are listed in Table 1.

**Table 1:** ICP-MS operating conditions and data acquisition parameters

Operation condition	
Nebuliser	Quartz concentric (Micromist) 400 μL/min
Spray chamber	Scott-type double-pass water cooled
Cell geometry	Octopole
Sampling cone	Nickel, 1.0 mm orifice
Skimmer cone	Nickel, 0.4 mm orifice
RF power	1400–1500 W
Reflected power	<10 W
He mode (collision cell mode)	
Plasma gas flow	15 L/min
Nebuliser gas flow	0.95–1.00 L/min
Auxiliary gas flow	0.99 L/min
He gas flow	3.5 mL/min
Expansion stage	2.0 mbar
Intermediate stage	2.0 × 10^−4^-3.0 × 10^−4^mbar
Analyzer stage	1.0 × 10^−4^-2.0 × 10^−4^mbar
Octopole bias	−18 V
Quadrupole bias	−16 V
Acquisition parameters	
Mass range	2-260a.m.u
Number of channels	500
Dwell time	300 ms
Number of sweeps	500
Total acquisition time	14.6400 s

The analysis of mercury (Hg) was analyzed by mercury analyzer (MA-2, Nippon Instrument Co., Tokyo, Japan) using 50 mg of decoction sample. Additive aluminum oxide (BHT®, Nippon Instruments Corp., Tokyo, Japan) was placed in an analytical boat with sample. And then additive BHT was covered and additive calcium hydroxide and sodium carbonate (MHT®, Nippon Instruments Corp., Tokyo, Japan) was filled on the boat. The mercury was decomposed by heating to high temperatures and vaporized, captured, and concentrated on the collector surface made of a multi-porous substance that was coated with gold. And then, it was released into an atomic absorption measuring device and measured at 253.7 nm by mercury analyzer. Five external working standards of Hg were prepared in a concentration range from 0 to 20 μg/kg by diluting Hg standard stock solution (994 mg/L, Kanto Soka, Japan) with 0.001% L-cysteine solution (98%, Nacalai Tesque Inc., Kyoto, Japan). The operation parameters of mercury analyzer are listed in Table 2.

**Table 2:** Mercury analyzer operating conditions

Parameter	Condition
Mode selector	Standard: 1, Sample : 2
Heating mode	Two available modes
Gas washing bottle	Buffer: H_2_O = 1:1(v/v)
Flow meter	0.5 L/min
Sample heating furnace H1	Mode 1 : 600 °C(2 min), Mode 2 : 800 °C(4 min)
Decomposing furnace H2	Heated at 850 °C
Mercury collector H3	About Heated at 700 °C
Carrier gas	Purified dry air
Additive	Standard: unnecessary, Sample: B + S + B + M^a^

^a^M: Sodium carbonate anhydrous: Calcium hydroxide = 1: 1 (v/v); B: Aluminum oxide anhydrous; S: Sample

### Quality assurance

Several parameters were evaluated for the validation of the analytical method followed, for determination of As, Cd, Pb and Hg in decoctions. The parameters were linearity, precision, accuracy, limit of detection (LOD), and limit of quantification (LOQ). For the linearity, linear regression coefficients (r2) should be >0.999 for As, Cd, Pb and Hg in every analytical batch. The accuracies of the measurements were assessed using NIST 1547 (peach leaves), NIST 1573a (tomato leaves) and Nist 1568b (rice flour) from National Institute of Standards and Technology (Gaithersburg, MD, USA) as Certified Reference Material. The precision of analytical procedure was usually expressed as the variance or the coefficient of variation (CV) of a series of measurements. The precision was evaluated using a relative standard deviation of five repeated determination of one sample [16]. The recovery percentages and percent coefficient of variation (CV%) are shown in Table 3. The capability of the method was estimated through the determination of the detection limits of every element studied. The limits of detection (LOD) and limits of quantification (LOQ) were calculated with three and ten times the standard deviation of the blank divided by the slope of the analytical curve, respectively [17].

**Table 3:** LOD, LOQ, precision and recovery for 4 heavy metals analyzed

Element	Reference Value	Found	Recovery (%)	Precision (CV%)	LOD	LOQ
	Mean ± SD (mg/kg)					
AS	0.285 ± 0.014^a^	0.298 ± 0.007	104.7	2.5	0.004 μg/kg	0.014 μg/kg
Cd	1.52 ± 0.04^b^	1.462 ± 0.005	96.2	0.3	0.003 μg/kg	0.009 μg/kg
Pb	0.87 ± 0.03^c^	0.703 ± 0.021	80.9	2.9	0.079 μg/kg	0.264 μg/kg
Hg	0.034 ± 0.004^b^	0.031 ± 0.003	100.0	4.7	0.017 mg/kg	0.057 mg/kg

^a^Rice flour Certified Reference Material (NIST 1568b), ^b^Tomato leaves Certified Reference Material (NIST 1573a), ^c^Peach leaves Certified Reference Material (NIST 1547), LOD: limits of detection, LOQ: limits of quantification

### Risk assessment

According to the recommendations from The Joint FAO/WHO Expert Committee on Food Additives [18] the risk assessment for contamination of the heavy metals was conducted by comparing the percentage value of provisional tolerable weekly intake (PTWI). PTWI value was converted from the Average Daily Dose (ADD, mg/kg/day) which was computed according to Eq. (1).

1 $$ \mathrm{ADD} = \left(\mathrm{CH}\times \mathrm{ID}\times \mathrm{EF}\times \mathrm{ED}\right)/\left(\mathrm{BW}\times \mathrm{AT}\right) $$

where ADD is the average daily dose (mg/kg/day), CH is the concentration of toxic metals (mg/kg), ID is the ingestion dose (mL/day), EF is the exposure frequency (day/year), ED is the exposure duration (60 year), BW is the body weight (kg), and AT is the average life span of Korean people (80 years). The exposure calculation was based on the following assumption; the ingestion of a 100 mL decoction twice a day to a 60 kg adult for 30 days performed 3 times in a year. Toxic metal concentrations for ingestion were set for two cases, a mean value and a 95th percentile value respectively.

### Analysis of residual agricultural chemicals

#### Determination of concentrations of 33 pesticides

Thirty three pesticides were analyzed using a protocol for multi class pesticide multiresidue methods proposed by ministry of food drug safety (MFDS) in Korean [19]. Gas chromatography/nitrogen phosphorous detector (GC/NPD, HP6890N, Agilent Technologies, USA) and GC/micro electron capture detector (GC/μECD, HP6890N, Agilent Technologies, USA) were used for analysis of 1 herbicides, 1 acaricides, 18 insecticides and 13 fungicides respectively (Additional file 1: Table S2). A 50 ml of distilled water was added to 20 g of decoction, and then the diluted decoction was extracted with 100 mL of acetonitrile using rotary shaker for 1 h. The homogenized decoction was filtered using a qualitative filter paper with 18.5 cm diameter (Ahlstrom Filtration LLC, Mt. Holly Springs, PA, USA), and then the filtrates were vigorously shaken in a milk bottle containing 10 g of sodium chloride. A 10 mL aliquot of the upper layer (acetonitrile layer) was then taken and evaporated to solid dryness in a water bath at 40 °C. The SPE florisil cartridges were used for purification process. The purified eluates were evaporated to solid dryness using a nitrogen evaporator at 40 °C. The dried extracts were redissolved in 2 mL of acetone-hexane (2:8, v/v) and prepared as test solution. Finally, 33 pesticides were analyzed using DB-5 and DB-1701 capillary dual column and detected by GC/NPD, GC/μECD and GC/MSD. The analytical conditions of the instruments are shown in Table 4.

**Table 4:** Analytical conditions of GC/NPD, GC/μECD and GC/MSD

Parameter	GC/NPD	GC/μECD	GC/MSD
Column	DB-1701^a^ DB-5^b^	DB-1701 DB-5	DB-5 MS^c^
Gas flow	N_2_ (1.4 mL/min) Air (60 mL/min) H_2_ (3.5 mL/min)	N2 (1 mL/min)	N2 (1 mL/min)
Injection port temperature	250 °C	230 °C	230 °C
Detector temperature	325 °C	280 °C	
Oven temperature	110 °C (1 min)-15 °C/min 200 °C (10 min)-20 °C/min 280 °C (17 min)	150 °C (0.5 min)-30 °C/min 190 °C (0.2 min)-1 °C/min 280 °C (11 min)	100 °C (2 min)-10 °C/min- 320 °C (5 min)

^a^30 m × 0.32 mm × 0.25 m; ^b^30 m × 0.32 mm × 0.25 m; ^c^30 m × 0.25 mm × 0.25 m

### Quality assurance

Quantitative analysis was conducted using an external standard (DB-5 capillary column). The linearity of each pesticide was established by plotting a GC response area versus concentration. The calibration curve was obtained by analyzing the pesticides at three different levels. The correlation coefficient (R2) was found to be ≥0.9992. All pesticides presented a linear behavior in the standard concentration range of 0.05–4.00 mg/kg. The limit of quantification (LOQ) was estimated at the lowest concentration of pesticide injected that yielded a signal/noise ratio of three (S/N) ≥3 for each pesticide, while the fortification concentration giving an S/N ≥10 was considered as the limit of quantification (LOQ). The LOD and LOQ for the test pesticides were in the range of 0.003–0.097 mg/kg and 0.009–0.293 mg/kg (Table 5). To assess the accuracy of the presented method, the recovery tests for 33 pesticides were conducted at three different conditions, 0.5 mg/kg, 1.0 mg/kg and 2.0 mg/kg (Additional file 1: Table S3).

**Table 5:** Linearity of calibration curve, LOD, and LOQ of pesticides

Pesticides	Regression equation	R2	^c^LOD mg/kg)	^d^LOQ (mg/kg)
p,p'-DDD	y^b^ = 374844.59x^a^-1258.40	1.0000	0.005	0.016
p,p'-DDE	y = 389791.58x-2526.36	1.0000	0.007	0.021
o,p'-DDT	y = 229348.05x-12990.11	0.9995	0.065	0.196
p,p'-DDT	y = 254190.24x-16171.13	0.9996	0.045	0.136
Bifenthrin	y = 85403.71x-1585.67	0.9999	0.030	0.091
Chlorfenapyr	y = 393884.32x-6112.32	1.0000	0.018	0.053
Chlorothalonil	y = 475988.22x-13117.75	0.9999	0.031	0.094
Cyhalothrin	y = 273407.08x-11711.69	0.9998	0.042	0.129
Cypermethrin	y = 146439.59x-7670.04	0.9995	0.060	0.181
Dieldrin	y = 474247.23x-4668.10	1.0000	0.007	0.020
α-Endosulfan	y = 435974.27x-2681.56	1.0000	0.011	0.034
β-Endosulfan	y = 411096.17x-3877.12	1.0000	0.015	0.045
Endosulfan Sulfate	y = 330896.35x-6530.19	0.9999	0.029	0.089
Fenarimol	y = 353478.59x-15843.60	0.9996	0.052	0.159
Fenpropathrin	y = 102000.65x-3871.97	0.9998	0.042	0.127
Hexaconazole	y = 189804.39x-19048.19	0.9999	0.033	0.101
Isoprothiolane	y = 102841.63x-4375.38	0.9996	0.051	0.154
Kresoxim-methyl	y = 82893.05x-1863.47	0.9999	0.026	0.080
Methoxychlor	y = 174878.92x-5581.06	0.9998	0.055	0.166
Pendimethalin	y = 95284.06x-766.05	0.9999	0.038	0.115
Procymidone	y = 48823.89x + 1832.31	0.9999	0.077	0.234
Tetradifon	y = 305112.99x-9039.28	0.9998	0.050	0.151
Thifluzamide	y = 290010.38x-5561.51	1.0000	0.012	0.035
Tolylfluanid	y = 269855.77x-12090.08	0.9999	0.022	0.067
Triadimefon	y = 190757.09x-3508.23	1.0000	0.022	0.066
Triflumizole	y = 142356.47x-8256.12	0.9992	0.097	0.293
Cyprodinil	y = 205.34x-2.68	1.0000	0.045	0.138
Iprobenfos	y = 1540.77x-171.09	0.9999	0.074	0.225
Napropamide	y = 51.25x-1.22	1.0000	0.036	0.109
Tebuconazole	y = 80.37x-8.08	1.0000	0.025	0.076
Tebufenpyrad	y = 121.95x-2.61	1.0000	0.044	0.132
Triadimenol	y = 91.36x-5.56	0.9999	0.054	0.162
Triazophos	y = 1540.77x-217.58	1.0000	0.003	0.009

^a^y = peak area, ^b^x = concentration of the respective compounds, ^c^LOD = 3.3 × δ/S, ^d^LOQ =10 × δ/S (δ : standard deviation, S : the individual slope in calibration curves)

### Analyses of sulfites

#### Determination of sulfites

Analysis of sulfites was carried out using the Monier-Williams Method [20]. This method measures free sulfite plus reproducible portion of bound sulfites, such as carbonyl addition products, in sample. This method is applicable of determination of ≥10 ppm sulfites in foods. 50 g of decoction samples were mixed with 100 mL 5% ethanol (99.9%, v/v, Fisher, Fair Lawn, Japan). Apparatus and water were deoxygenated with N_2_ flow at 200 ± 10 mL/min for 15 min. Prepared test portion was introduced into the three-neck round bottom distillation flask with 400 mL of water. In the receiving vessel 30 mL of H_2_O_2_ (30%, v/v, Junsei, Tokyo, Japan), previously titrated to a yellow end point with 0.01 N NaOH (Wako, Osaka, Japan), was placed. 90 mL 4 N HCl (35.0 ~ 37.0%, v/v, Wako, Japan) were added to the flask and the distillation was completed in 1 h 45 min. In this way, sulphurous anhydride was distilled and converted to sulphuric acid by reaction with of H_2_O_2._ The sulfuric acid was titrated against 0.01 N NaOH with methyl red (Acros, New Jersey, USA) as indicator up to a yellow endpoint that persisted for ≥20 s.

### Quality assurance

The accuracy and precision of the method as a routine analysis method was estimated through the FAPAS T20100QC (Meat) from Food Analysis Performance Assessment Scheme (Sand Hutton, York, United Kingdom) as Reference Material. The accuracy and precision of sulfites determination by the Monier-Williams Method was calculated as the recovery and CV % from the analyses of five replicates as shown in Table 6.

**Table 6:** Precision and recovery for the Sulfur Dioxide analyzed

Assigned Value (mg/kg) Range for ∣z∣ ≤ 2	Found	Recovery (%)	Precision (CV%)
	Mean ± SD (mg/kg)		
453 (375–531)^a^	497.6 ± 3.2	109.8	0.6

^a^Meat Reference Material (FAPAS T20100QC)

### Statistical analysis

All data are expressed as average value of concentration and standard deviation as well as their distribution range. Based on the their main clinical indications, the decoction samples were classified into 9 subgroups according to the 10th revision of the International Statistical Classification of Diseases and Related Health Problems after slight modification [21] The differences of average among 9 groups were assessed by one-way analysis of variance followed by a paired Student’s *t*-test. Differences with a *P* < 0.05 were considered significant.

## Results

### Concentration of heavy metals

One hundred fifty two samples (98.1%) of 155 decoctions contained at least one of three heavy metals as over detectable levels; As (96.1%), Cd (97.4%), and Pb (90.3%), but not for Hg (0.0%). Their average concentrations were 77.0 ± 79.7 ug/kg (range 0 to 582.4) for As, 20.4 ± 23.7 ug/kg (range 0 to 219.0 ug/kg) for Cd, and 68.8 ± 76.5 ug/kg (range 0 to 631.7 ug/kg) for Pb respectively. The 95th percentile concentration was approximately 5-folds of average value (304.4 ± 121.1 ug/kg for As, 90.1 ± 56.2 ug/kg for Cd and 285.8 ± 144.1 ug/kg for Pb respectively) (Table 7).

**Table 7:** Concentration of heavy metals, sulfur dioxide and pesticides in herbal decoctions

Clarification (Sample N.)	Average ± SD (Range value, ug/kg, but mg/kg for sulfites)					
	As	Cd	Pb	Hg	Pesticides	Sulfites
Nutritional disorders (41)	82.9 ± 97.6 (1.3–582.4)	17.8 ± 12.6 (2.9–41.2)	66.5 ± 100.4 (0.0–631.7)	ND^a^	ND	17.6 (1 sample)
Respiratory disorders (36)	78.5 ± 84.7 (0.0–374.4)	25.4 ± 39.5 (0.0–219.0)	75.8 ± 71.6 (0.0–242.8)	ND	ND	ND
Digestive disorders (22)	67.3 ± 67.2 (0–106.9)	14.9 ± 11.9 (0–22.1)	67.1 ± 55.8 (0–121.6)	ND	ND	ND
Genitourinary disorders (12)	82.6 ± 85.3 (13.5–245.9)	18.2 ± 22.9 (1.7–86.2)	65.7 ± 78.1 (0.5–25.3)	ND	ND	ND
Muscular disorders (12)	118.6 ± 59.6 (7.4–229.4)	36.3 ± 21.4 (3.2–93.9)	107.2 ± 51.0 (21.0–04.7)	ND	ND	ND
Gynecological disorders (10)	117.1 ± 74.4 (0.0–237.1)	27.6 ± 14.3 (0.0–45.9)	118.7 ± 95.7 (0–308.4)	ND	ND	ND
Psychiatric disorders (10)	30.2 ± 25.7 (0.0–91.5)	11.7 ± 13.1 (0.0–39.9)	21.2 ± 35.2 (0.0–119.2)	ND	ND	ND
Circulatory disorders (6)	50.9 ± 34.4 (14.6–112.6)	16.6 ± 16.7 (2.9–46.8)	43.5 ± 28.3 (0.0–79.8)	ND	ND	ND
Skin disorders (6)	58.9 ± 42.5 (4.0–129.7)	20.0 ± 12.8 (0.4–38.1)	52.8 ± 27.2 (9.7–81.7)	ND	ND	17.6 (1 sample)
Total (155)	96.1%^b^	97.4%	90.3%	0%	0%	0.7%
	77.0 ± 79.7	20.4 ± 23.7	68.8 ± 76.5	-	-	-
	(0.0–582.4)	(0.0–219.0)	54 (0.0–631.7)	-	-	-
95th percentile	304.4 ± 121.1	90.1 ± 56.2	285.8 ± 144.1			

^a^ND: Not detected in any sample over detectable level; ^b^Detection rate for each heavy metal

Among 9 subgroups of samples based on the their clinical main indications by ICD-10, decoctions for psychiatric disorders showed the lowest concentration (approximately 30 to 57% of whole sample) for As, Cd and Pb while samples for muscular disorders showed the highest value (approximately 150 to 180% of whole sample). No significant difference was however observed among the groups for their average concentrations (Table 7).

### Concentration of pesticides and sulfites

None of herbal drug sample showed the detectable level for any kinds of 33 pesticides. Regarding the content of residual sulfites, only one decoction sample showed over limit of detection (10 mg/kg), as a 17.6 mg/kg (Table 7).

### PTWI values of heavy metals

Average weekly doses (μg/kg/week) of total samples were 0.34, 0.09, and 0.03 for As, Cd and Pb, which were correspondent to 2.2, 1.3 and 1.2% of PTWI guided by JECFA. Meanwhile their 95th percentile values were 1.33 (8.9%), 0.39 (5.6%), and 1.26 (5.0%) for As, Cd and Pb respectively (Table 8). No significant difference was observed among the 9 subgroups (data not shown).

**Table 8:** ADD and PTWI values of heavy metals in herbal decoctions

Contents	As	Cd	Pb	Hg
Provisional tolerable weekly intake (PTWI, μg/kg/week)^a^	15	7	25	5
Average daily dose (ADD, μg/kg/day)	0.05	0.01	0.04	0
ADD for 95th percentile (μg/kg/day)	0.19	0.06	0.18	0
Average weekly dose (AWD, μg/kg/week)	0.34	0.09	0.30	0
Ratio to PTWI	2.2%	1.3%	1.2%	0%
AWD for 95th percentile (μg/kg/week)	1.33	0.39	1.25	0
Ratio to PTWI	8.9%	5.6%	5.0%	0%

^a^[18, 32]

## Discussion

In term of the scientific requirement for safety and efficacy of herbal products, the quality control of herbal resources is priority, and then the assessment of adulteration or contamination with undeclared materials including hazardous substance is an overriding consideration [22]. Therefore, WHO had developed guidelines for assessing quality of herbal medicines, and particularly considered the potential risk of contaminants from the soil or other environmental sources [14]. In this study, we have monitored the residual levels of four heavy metals, 33 pesticides and sulfites in herbal decoctions, which can be contaminated in the process of cultivation, harvesting and storage.

From the measurement of the four residual heavy metals, we found that 98.1% of samples contained the detectable levels of at least As, Cd, or Pb respectively. This detection rate is notably higher than other studies in UAS, which presented the contamination of As, Cd, Pb or Hg in 20.0% of Ayurvedic herbal products and 19.4% of Hispanic herbal remedies [23, 24]. This big gap in the prevalence of heavy metal contamination between our study and these reports might result from the drastic difference of detectable levels. In general, traditional Indian medicines are known to use commonly the metal-contained herbal drugs [25], and then about 1000 fold higher concentration of heavy metals comparing to our study was observed in above two studies [23, 24]. In our study, the residual Hg was not detected in any sample; meanwhile the LOD of Hg was much higher than other three heavy metals.

In case of exceeding intake, heavy metals poisoning can be induced. Above four heavy metals rank among the priority metals that are of public health significance due to their high degree of toxicity, which can cause multiple organ damage, even at lower levels of exposure [25]. The herbal drug-derived poisoning of heavy metals are frequently reported, likely hemolytic anemia by arsenic intoxication [26], congenital lead poisoning [27], and mercury toxicity following herbal preparations [28]. Moreover, As, Cd, Pb and Hg are carcinogenic toxic metals [29, 30]. Accordingly, their contents in herbal products are regulated by governments. Our results showed that As, Cd and Pb contents were 2.6, 6.7 and 1.4% of the maximum allowable limits for herbal medicinal preparation (As < 3 mg/kg, Cd < 0.3 mg/kg, Pb < 5 mg/kg, and Hg < 0.2 mg/kg) by Korean MFDS [19].

The final utilizing form of medicinal herbs is generally the decoction especially in East Asia countries; however no guide about the heavy metal contamination exists for herbal decoction. When we compare the beverages using vegetable, tea or ginseng (Cd < 0.1 mg/kg and Pb < 0.3 mg/kg) described in the Korean Food Standard Codex [19], our data are near to 20% of the maximum allowable limits for Cd and Pb respectively. The concentrations of those heavy metals were very wide, thus we considered the cases of the top ranking samples for heavy metals. The 95th percentile concentration was approximately 4-folds of average value (0.3 mg/kg for As, 0.09 mg/kg for Cd and 0.3 mg/kg for Pb respectively), which is still less than the guideline for above beverages. In addition, because toxic metals are cumulative poisons, the JECFA recommends comparing the percentage value of PTWI for the individual heavy metals [31]. The PTWI values less than 2.2% for total samples and 8.9% for 95th percentile respectively. This result would indicate that the herbal decoctions generally contain the safe range of heavy metals, regarding As, Cd, Pb, and especially Hg. We herein adapted PTWI value of As (15 μg/kg/week). In fact, JECFA however withdrew this PTWI value in 2010 because it was believed to be inappropriate [32]. High levels of toxic metals can sometimes occur in Chinese or Indian herbal medicines when they are used as active ingredients [33], while those cases were absent in our study.

On the other hand, the contamination with residual pesticides or sulfites in herbal remedies is another consideration. The major source of above toxic metal in herbal preparation is the environmental contamination including the soil, water and air [34], while contaminations of the residual pesticides result from mainly in the process of cultivation or harvesting [35]. Although the today’s proper use of pesticides is safe and can improve the yield and quality of the agricultural products, there are many concerns about the potential risks associated with pesticide use [36]. Therefore, likely many countries Korean government have restricted the usage of these pesticides establishing tolerances or maximum residue limits (MRLs) in herbal materials [37]. In our study no residual pesticide was detected in 155 decoction samples. In addition, our data revealed that only one sample (decoction of *Bojungikkitang*, 补中益气汤) contained the detectable level of sulfites (≥10 mg/kg). Sulfites are used as food preservative and can naturally occur in some foods [38], however intake of excess sulfites has been reported to induce a various adverse effects including dermatitis, hypotension, diarrhoea or asthmatic reactions, in especially sensitive individuals [39, 40]. Codex Alimentarius Commission (CAC) recommended that products with sulfites ≥10 mg/kg should always be declared [41], and Korean movement has regulated it with the maximum residue limits (MRLs) of sulfites as 30 mg/kg in medicinal herbs since 2009 [37]. *Bojungikkitang* (补中益气汤) is a typical formula supporting the *Qi* of digestive track, which composed of 8 medicinal herbs (*Astragali membranaceus* Bunge, *Panax ginseng* C.A. Meyer, *Atractylodes japonica* Koidzumi, *Glycyrrhiza uralensis* Fischer, *Angelica gigas* Nakai, *Citrus unshiu* Markovich, *Cimicifuga heracleifolia* Komarov and *Bupleurum falcatum* Linne) The exact reason for the high level of sulfites (17.6 mg/kg) in the decoction was unknown.

The quantity of contaminants can be changed in manufacturing process for final products. A study presented the significantly low transfer rates of toxic metals (10.5% for As, 4.1% for Cd, 4.3% for Pb, and 2.7% for Hg) after decoction process of herbal formulae [42]. One study reported a 5.3% detection rate of residual pesticides and 0.9% excess MRLs rate among 1565 medicinal herbs [43], and another study presented the 12.5% excess MRLs rate among136 medicinal herbals [44] in Korea. These data are notably different with our results in current study. One study showed that the boiling process reduces the contamination levels of unwanted contents including heavy metals [45], which is able to explain our finding.

## Conclusions

Taken together, our study presents the current status of herbal decoctions regarding the contamination with heavy metals, pesticides and sulfites, which are in the safe range in Korea. This data would provide a reference to the research field of herbal preparation.

## Additional file

Additional file 1:
**Table S1.** List of 155 herbal formulae and their main compositions. **Table S2**. Pesticides analyzed in herbal decoctions. **Table S3**. Recovery for 33 pesticides at three concentration levels. (DOC 231 kb)

## Abbreviations

ADD: Average daily dose
AWD: Average weekly dose
CAC: Codex Alimentarius Commission
CV%: Percent coefficient of variation
CV: Coefficient of variation
GC/NPD: Gas chromatography/nitrogen phosphorous detector
GC/μECD: GC/micro electron capture detector
LOD: Limit of detection
LOQ: Limit of quantification
MRLs: Maximum residue limits
ND: Not detected in any sample over detectable level
PTWI: Provisional tolerable weekly intake
R2: Correlation coefficient
S/N: Signal/noise ratio of three

## References

[1] WHO (2013). WHO traditional medicine strategy.

[2] Yoo TW, Kim BI, Kim JB (2007). The survey for the actual condition of drug medication and development of health care cost associated with toxic liver injury in Korea: a multicenter study for the detection and the development of nationwide reporting system of toxic liver injury. Korean J Hepatol.

[3] Xu J, Yang Y (2009). Traditional Chinese medicine in the Chinese health care system. Health Policy.

[4] Barnes PM, Bloom B, Nahin RL (2008). Complementary and alternative medicine use among adults and children: United States, 2007. Natl Health Stat Report.

[5] Jeong TY, Park BK, Cho JH (2012). A prospective study on the safety of herbal medicines, used alone or with conventional medicines. J Ethnopharmacol.

[6] Street RA, Stirk WA, Van Staden J (2008). South African traditional medicinal plant trade-Challenges in regulating quality, safety and efficacy. J Ethnopharmacol.

[7] Fong HH (2002). Integration of herbal medicine into modern medical practices: issues and prospects. Integr Cancer Ther.

[8] Li SP, Zhao J, Yang B (2011). Strategies for quality control of Chinese medicines. J Pharm Biomed Anal.

[9] Sahoo N, Manchikanti P, Dey S (2010). Herbal drugs: standards and regulation. Fitoterapia.

[10] Zhang J, Wider B, Shang H, Li X, Ernst E (2012). Quality of herbal medicines: challenges and solutions. Complement Ther Med.

[11] Sarma H, Deka S, Deka H, Saikia RR (2011). Accumulation of heavy metals in selected medicinal plants. Rev Environ Contam Toxicol.

[12] Oh CH (2009). Monitoring of residual pesticides in herbal drug materials of Korea and China. Bull Environ Contam Toxicol.

[13] Kan WL, Ma B, Lin G (2011). Sulfur fumigation processing of traditional chinese medicinal herbs: beneficial or detrimental?. Front Pharmacol.

[14] WHO (2007). WHO guidelines for assessing quality of herbal medicines with reference to contaminants and residues.

[15] Seo CS, Huang DS, Lee JK (2009). Concentration of heavy metals, residual pesticides and sulfur dioxide before/after a decoction. J Korean Oriental Med.

[16] Chudzinska M, Baralkiewicz D (2011). Application of ICP-MS method of determination of 15 elements in honey with chemometric approach for the verification of their authenticity. Food Chem Toxicol.

[17] Thompson M, Ellison SLR, Wood R (2002). Harmonized guidelines for single laboratory validation of methods of analysis (IUPAC Technical Report). Pure Appl Chem.

[18] JECFA (1997). Report of the Joint FAO/WHO consultation of food consumption and exposure assessment of chemicals.

[19] MFDS. Notification for Food Standards and Specifications (Number 2015-78). http://www.mfds.go.kr/index.do?mid=686&pageNo=4&seq=10086&cmd=v. Accessed 15 May 2016.

[20] Association of Analytical Communities (2000). AOAC Official Methods of Analysis. chapter 47. Monier-Williams AOAC official method (optimized method) 990.28.

[21] WHO. International Statistical Classification of Diseases and Related Health Problems −10 (ICD-10). http://apps.who.int/classifications/icd10/browse/2016/en. Accessed 15 May 2016.

[22] Posadzki P, Watson L, Ernst E (2013). Contamination and adulteration of herbal medicinal products (HMPs): an overview of systematic reviews. Eur J Clin Pharmacol.

[23] Saper RB, Kales SN, Paquin J (2004). Heavy metal content of ayurvedic herbal medicine products. JAMA.

[24] Levine M, Mihalic J, Ruha AM, French RN, Brooks DE (2013). Heavy metal contaminants in yerberia shop products. J Med Toxicol.

[25] Gogtay NJ, Bhatt HA, Dalvi SS, Kshirsagar NA (2002). The use and safety of non-allopathic Indian medicines. Drug Saf.

[26] Lee JJ, Kim YK, Cho SH (2004). Hemolytic anemia as a sequel of arsenic intoxication following long-term ingestion of traditional Chinese medicine. J Korean Med Sci.

[27] Tait PA, Vora A, James S, Fitzgerald DJ, Pester BA (2002). Severe congenital lead poisoning in a preterm infant due to an herbal remedy. Med J Aust.

[28] Soumya L, Pandalai, Brent W (2011). Morgan case files of the Emory university medical toxicology fellowship: inhalational mercury toxicity from a traditional Vietnamese product. J Med Toxicol.

[29] IARC. IARC monographs on the evaluation of carcinogenic risks to humans. http://monographs.iarc.fr/ENG/Classification/index.php. Accessed 15 May 2016.

[30] Sohn J, Lee C (2007). The risk assessment of hazard chemical in environment. J Korean Soc Environ Eng.

[31] Kim JA, Lee SH, Choi SH (2012). Heavy metal risk management: case analysis. Toxicol Res.

[32] JECFA. Short information documents for decision makers. http://www.who.int/ipcs/assessment/public_health/arsenic/en/. Accessed 15 May 2016.

[33] Ernst E (2002). Toxic heavy metals and undeclared drugs in Asian herbal medicines. Trends Pharmacol Sci.

[34] Ezeabara CA, Okanume OE, Emeka AN, Okeke CU, Mbaekwe EI (2014). Heavy metal contamination of herbal drugs: implication for human health-a review. Int J Trop Dis Health.

[35] Oh CH (2007). Multi residual pesticide monitoring in commercial herbal crude drug materials in South Korea. Bull Environ Contam Toxicol.

[36] Zhou P, Zhao Y, Li J (2012). Dietary exposure to persistent organochlorine pesticides in 2007 Chinese total diet study. Environ Int.

[37] MFDS. Notification for Food Standards and Specifications (Number 2009–35). http://www.mfds.go.kr/index.do?mid=686&seq=2435&cmd=v. Accessed 15 May 2016.

[38] Mischek D, Krapfenbauer-Cermak C (2012). Exposure assessment of food preservatives (sulphites, benzoic and sorbic acid) in Austria. Food Addit Contam.

[39] Zhang JB, Zhang H, Wang HL (2014). Risk analysis of sulfites used as food additives in China. Biomed Environ Sci.

[40] Vally H, Misso NL, Madan V (2009). Clinical effects of sulphite additives. Clin Exp Allergy.

[41] FAO/WHO. Codex general standard for the Labelling of Prepackaged Foods. CODEX STAN 1–1985. 2008.

[42] Kim DG, Lee SD, Yu IS, Jung K, Park SK (2015). Transfer rates of toxic metals during decoction preparation from hebal medicines and safety evaluation of the final decoction products. Food Sci Biotechnol.

[43] Choi YH, Park SK, Kim OH (2011). Pesticide residues monitoring of medicinal herbs in Seoul. Korean J Pesticide Sci.

[44] Lee AR, Jang S, Kim TH (2013). Monitoring of residual sulfur dioxide in herbal medicines distributed at domestic. Korean J Medicinal Crop Sci.

[45] Ting A, Chow Y, Tan W (2013). Microbial and heavy metal contamination in commonly consumed traditional Chinese herbal medicines. J Tradit Chin Med.

