# Supplementary material for: Monitoring heavy metals, residual agricultural chemicals and sulfites in traditional herbal decoctions
Source: BMC Complement Altern Med. 2017 Mar 14;17:154. doi: 10.1186/s12906-017-1646-y (PMC5348833; doi:10.1186/s12906-017-1646-y)
Supplement: Additional file 1: — Table S1. List of 155 herbal formulae and their main compositions. Table S2. Pesticides analyzed in herbal decoctions. Table S3. Recovery for 33 pesticides at three concentration levels. (DOC 231 kb) [file 12906_2017_1646_MOESM1_ESM.doc]

| **Supplementary Table 1. List of 155 herbal formulae and their main compositions** | |
| --- | --- |
| **Name of formula**  **(Chinese name)** | **Herbal composition** |
| ***From Oriental Clinic (89 formulae)*** | |
| 1. Dokhwaljihwangtang (独活地黄汤) | Rehmanniae Radix Preparata, Corni Fructus, Hoelen, Alismatis Rhizoma, Moutau Cortex |
| 2. Gungguijohyeoleum (芎归调血飮) | Leonuri Herba, Glycyrrhizae Radix, Cnidii Rhizoma, Cyperi Rhizoma, Angelicae Gigantis Radix |
| 3. Hyunbulikyungtang (玄附理经汤) | Linderae Radix, Cyperi Rhizoma, Aurantii nobilis Pericarpium, Zedoariae Rhizoma, Cinnamomi Cortex Spissus |
| 4. Joweseungcheongtang (调胃升淸汤) | Castaneae Semen, Coicis Semen, Platycadi Radix, Schizandrae Fructus |
| 5. Sibjeondaebotang1 (十全大补汤1) | Rehmanniae Radix Preparata, Astragal Radix, Paeoniae Radix, Angelicae Gigantis Radix, Cnidii Rhizoma |
| 6. Daeyoungjeon1 (大营煎1) | Paeoniae Radix, Angelicae Gigantis Radix, Cnidii Rhizoma, Cinnamomi Cortex Spissus, Glycyrrhizae Radix, Rehmanniae Radix Preparata, Lycii Fructus |
| 7. Ojeucksan1 (五积散1) | Atractylodis Rhizoma, Platycadi Radix, Dinelliae Tuber, Aurantii Fructus, Pachyma hoelen rumphius |
| 8. Gwakhyangjeonggisan1 (藿香正气散1) | Arecae Pericarpium, Agastachis Herba, Aurantii nobilis Pericarpium, Machili Cortex, Atractylodis Rhizoma Alba |
| 9. Yukmiojatang (六味五子汤) | Rehmanniae Radix Preparata, Dioscoreae Rhizoma, Corni Fructus, Lycii Fructus, Rubi Fructus |
| 10. Yeongsinhaedoktang (灵神解毒汤) | Osterici Radix, Araliae Cordatae Radix, Ledebouriellae Radix, Angelicae dahuricae Radix, Cnidii Rhizoma |
| 11. Guibitanggagam1 (归脾汤加减1) | Zizyphi Spinosi Semen, Longanae Arillus, Angelicae Gigantis Radix, Astragal Radix, Atractylodis Rhizoma Alba |
| 12. Bojungikkitanggagam1 (补中益气加减1) | Astragal Radix, Gingseng Radix, Liriopis Tuber, Hoelen Cum Radix, Lycii Fructus |
| 13. Talmoyebangtang (脱毛豫防汤) | Angelicae Gigantis Radix, Cnidii Rhizoma, Rehmanniae Radix Preparata, Asiasari Radix, Carthami Flos |
| 14. Kihyultang (气血汤) | Astragal Radix, Paeoniae Radix, Puerariae Radix, Gingseng Radix, Salviae Radix |
| 15. Danguichumtongtang (当归拈痛汤) | Puerariae Radix, Artemisiae capillaris Herba, Chaenomelis Fructus, Osterici Radix, Araliae Cordatae Radix |
| 16. Bojungikkitanggagam2 (补中益气加减2) | Astragal Radix, Atractylodis Rhizoma Alba, Aurantii nobilis Pericarpium, Cyperi Rhizoma, Glycyrrhizae Radix |
| 17. Ondamtang (温胆汤) | Gardeniae Fructus, Coptidis Rhizoma, Ponciri Fructus, Hoelen, Aurantii nobilis Pericarpium |
| 18. Bojungikkitang (补中益气汤) | Astragali membranaceus Bunge, Panax ginseng C.A. Meyer, Atractylodis Rhizoma Alba, Glycyrrhizae Radix, Angelicae Gigantis Radix, Aurantii nobilis Pericarpium |
| 19. Chungsimyeonjaeum (淸心莲子飮) | Nelumbinis Semen, Astragal Radix, Gingseng Radix, Glycyrrhizae Radix, Scutellariae Radix, Lycii Cortex Radicis |
| 20. Chongmyungtang (聪明汤) | Dioscoreae Rhizoma, Angelicae Gigantis Radix, Astragal Radix, Rehmanniae Radix Preparata, Aurantii nobilis Pericarpium, Alismatis Rhizoma |
| 21. Ojeucksan2 (五积散2) | Atractylodis Rhizoma, Aurantii nobilis Pericarpium, Machili Cortex, Platycadi Radix, Angelicae Gigantis Radix, Paeoniae Radix |
| 22. Hyangsayangyitang (香砂养胃汤) | Atractylodis Rhizoma Alba, Amomi Semen, Atractylodis Rhizoma, Machili Cortex, Aurantii nobilis Pericarpium, Pachyma hoelen rumphius |
| 23. Gamissangwhatang1 (加味双和汤1) | Astragal Radix, Angelicae Gigantis Radix, Lycii Fructus, Cassiae Cortex, Agastachis Herba, Cnidii Rhizoma, Paeoniae Radix |
| 24. Biyumtang (鼻炎汤) | Magnoliae Flos, Gleditsiae Fructus, Ponciri Fructus, Glycyrrhizae Radix |
| 25. Sungjangtang (成长汤) | Astragal Radix, Angelicae Gigantis Radix, Cnidii Rhizoma, Dendrobii Herba, Amomi Semen |
| 26. Pyungwisangasamultang (平胃散加四物汤) | Atractylodis Rhizoma, Aurantii nobilis Pericarpium, Glycyrrhizae Radix, Angelicae Gigantis Radix, Rehmanniae Radix Preparata |
| 27. Pibusoyangtang (皮肤搔痒汤) | Bupleuri Radix, Cinnamomi Ramulus, Paeoniae Radix, Cyperi Rhizoma, Cnidii Rhizoma |
| 28. Galgeunhaegitang1 (葛根解肌汤1) | Puerariae Radix, Angelicae tenuissimae Radix, Angelicae dahuricae Radix, Scutellariae Radix, Platycadi Radix |
| 29. Palmultanggagam1 (八物汤加减1) | Angelicae Gigantis Radix, Rehmanniae Radix Preparata, Paeoniae Radix, Cnidii Rhizoma, Astragal Radix |
| 30. Gejimahwanggakbantanggagam (桂枝麻黄各半汤加减) | Cinnamomi Cortex Spissus, Paeoniae Radix, Zingiberis Rhizoma, Zizyphi Fructus, Glycyrrhizae Radix, Ephedrae Herba |
| 31. Gamimahwangtang (加味麻黄汤) | Rehmanniae Radix Preparata, Glycyrrhizae Radix, Aurantii nobilis Pericarpium, Pachyma hoelen rumphius, Ephedrae Herba, Atractylodis Rhizoma Alba |
| 32. Gamisukjitang (加味熟地汤) | Rehmanniae Radix Preparata, Paeoniae Radix, Longanae Arillus, Puerariae Radix, Astragal Radix, Chaenomelis Fructus |
| 33. Gamihyangbujatang (加味香附汤) | Cyperi Rhizoma, Puerariae Radix, Cnidii Rhizoma, Sojae Semen Preparatum, Paeoniae Radix, Cimicifugae Rhizoma |
| 34. Yuldahansotang (热多寒少汤) | Puerariae Radix, Cimicifugae Rhizoma, Nelumbinis Semen, Dioscoreae Rhizoma, Liriopis Tuber, Asparagi Radix |
| 35. Gamibojungikkitang1 (加味补中益气汤1) | Astragal Radix, Gingseng Radix, Atractylodis Rhizoma Alba, Glycyrrhizae Radix, Angelicae Gigantis Radix, Aurantii nobilis Pericarpium, Bupleuri Radix, Cimicifugae Rhizoma, Crataegi Fructus, Massa Medicata Fermentata, Hordei Fructus Germinatus |
| 36. Gamimacmundongtang (加味麦门冬汤) | Pachyma hoelen rumphius, Atractylodis Rhizoma Alba, Liriopis Tuber, Asparagi Radix, Bupleuri Radix, Scutellariae Radix, Anthrisci sylvestris Radix, Platycadi Radix, Aurantii Fructus, Fritillariae Bulbus, Mori Cortex Radicis, Codonopsis Radix, Armeniacae Semen, Glycyrrhizae Radix, Nepetae Herba, Ledebouriellae Radix, Schizandrae Fructus, Coptidis Rhizoma |
| 37. Gamigungjihyangsosan (加味芎芷香苏散) | Cyperi Rhizoma, Atractylodis Rhizoma, Puerariae Radix, Aurantii nobilis Pericarpium, Perillae Herba, Nepetae Herba, Ledebouriellae Radix, Cnidii Rhizoma, Osterici Radix, Angelicae dahuricae Radix, Glycyrrhizae Radix, Zingiberis Rhizoma |
| 38. Gamissangwhatang2 (加味双和汤2) | Rehmanniae Radix Preparata, Angelicae Gigantis Radix, Cnidii Rhizoma, Paeoniae Radix, Astragal Radix, Cassiae Cortex, Glycyrrhizae Radix, Anemarrhenae Rhizoma, Phellodendri Cortex, Osterici Radix, Ledebouriellae Radix |
| 39. Gamibojungikkitang2 (加味补中益气汤2) | Cnidii Rhizoma, Rehmanniae Radix Preparata, Schizandrae Fructus, Crataegi Fructus, Massa Medicata Fermentata, Hordei Fructus Germinatus |
| 40. Gamiseungmagalgeuntang (加味升摩葛根汤) | Puerariae Radix, Paeoniae Radix, Cimicifugae Rhizoma, Glycyrrhizae Radix, Crataegi Fructus, Betula Platyphylla, Lonicerae Flos, Bardanae Fructus, Cornu Bubali, Nepetae Herba, Ledebouriellae Radix, Zingiberis Rhizoma |
| 41. Dokhwalsokdantang (独活续断汤) | Araliae Cordatae Radix, Phlomidis Radix, Angelicae Gigantis Radix, Aurantii nobilis Pericarpium, Hoelen, Cnidii Rhizoma, Paeoniae Radix, Eucommiae Cortex, Cinnamomi Cortex Spissus, Ledebouriellae Radix, Asiasari Radix, Achyranthis Radix, Glycyrrhizae Radix |
| 42. Gamijaeumgeonbitang (加味滋阴建脾汤) | Atractylodis Rhizoma Alba, Pachyma hoelen rumphius, Dinelliae Tuber, Aurantii nobilis Pericarpium, Angelicae Gigantis Radix, Rehmanniae Radix Preparata, Zingiberis Rhizoma, Gingseng Radix, Paeoniae Radix, Liriopis Tuber, Polygalali Radix, Rhemanniae Radix, Zizyphi Fructus, Cnidii Rhizoma, Glycyrrhizae Radix, Amomi Semen, Zizyphi Spinosi Semen |
| 43. Hyeongbangpaedoksan (荆防败毒散) | Osterici Radix, Araliae Cordatae Radix, Bupleuri Radix, Anthrisci sylvestris Radix, Nepetae Herba, Ledebouriellae Radix |
| 44. Gamipyeongwisan (加味平胃散) | Atractylodis Rhizoma, Aurantii nobilis Pericarpium, Machili Cortex, Platycadi Radix, Aurantii Fructus, Glycyrrhizae Radix |
| 45. Magyeum (麻桂飮) | Angelicae Gigantis Radix, Ephedrae Herba, Cinnamomi Cortex Spissus, Glycyrrhizae Radix, Aurantii nobilis Pericarpium, Zingiberis Rhizoma |
| 46. Ssanghwatang1 (双和汤1) | Paeoniae Radix, Angelicae Gigantis Radix, Cnidii Rhizoma, Rehmanniae Radix Preparata, Cinnamomi Cortex Spissus, Glycyrrhizae Radix |
| 47. Gamiyukmitang (加味六米汤) | Rehmanniae Radix Preparata, Rhemanniae Radix, Lycii Fructus, Corni Fructus, Alismatis Rhizoma, Hoelen |
| 48. Sibimijihwangtang (十二味地黄汤) | Rehmanniae Radix Preparata, Corni Fructus, Pachyma hoelen rumphius, Alismatis Rhizoma, Moutau Cortex, Lycii Cortex Radicis, Scrophulariae Radix, Lycii Fructus, Rubi Fructus, Plantayinis Semen, Nepetae Herba, Ledebouriellae Radix |
| 49. Gamibojungikkitang3 (加味补中益气汤3) | Astragal Radix, Gingseng Radix, Atractylodis Rhizoma Alba, Glycyrrhizae Radix, Angelicae Gigantis Radix, Aurantii nobilis Pericarpium, Cimicifugae Rhizoma, Bupleuri Radix, Anemarrhenae Rhizoma, Phellodendri Cortex, Liriopis Tuber, Schizandrae Fructus, Cyperi Rhizoma, Longanae Arillus, Dinelliae Tuber, Pachyma hoelen rumphius, Machili Cortex, Saussureae Radix, Amomi Semen, Amomi Cardamomi Fructus, #N/A, Zingiberis Rhizoma, Zizyphi Fructus |
| 50. Gamisamultang (加味四物汤) | Angelicae Gigantis Radix, Cnidii Rhizoma, Paeoniae Radix, Rehmanniae Radix Preparata, Pachyma hoelen rumphius, Glycyrrhizae Radix, Atractylodis Rhizoma Alba, Astragal Radix, Cinnamomi Cortex Spissus, Zizyphi Spinosi Semen, Cyperi Rhizoma |
| 51. Yunggamgangmisinhatang (苓甘姜味辛夏汤) | Dinelliae Tuber, Hoelen, Glycyrrhizae Radix, Schizandrae Fructus, Zingiberis siccatum Rhizoma, Asiasari Radix |
| 52. Guibitanggagam2 (归脾汤加减2) | Longanae Arillus, Zizyphi Spinosi Semen, Angelicae Gigantis Radix, Hoelen cum Pini Radix, Atractylodis Rhizoma Alba, Paeoniae Radix, Aurantii nobilis Pericarpium, Polygalali Radix, Cnidii Rhizoma, Rehmanniae Radix, Gingseng Radix, Saussureae Radix, Amomi Semen, Glycyrrhizae Radix, Menthae Herba, Liriopis Tuber, Bupleuri Radix, Gardeniae Fructus, Platycadi Radix, Bambusae Calulis in Taeniam, Ponciri Fructus, Aurantii Fructus |
| 53. Taeeumjowitanggami (太阴调胃汤加味) | Castaneae Semen, Coicis Semen, Raphani Semen, Platycadi Radix, Ephedrae Herba, Liriopis Tuber, Schizandrae Fructus, Acori Graminei Rhizoma, Rhei Rhizoma |
| 54. GamiSsangkumtang (加味双金汤) | Atractylodis Rhizoma, Aurantii nobilis Pericarpium, Pachyma hoelen rumphius, Massa Medicata Fermentata, Hordei Fructus Germinatus, Glycyrrhizae Radix, Agastachis Herba, Rehmanniae Radix Preparata, Angelicae Gigantis Radix, Cnidii Rhizoma, Paeoniae Radix, Cinnamomi Ramulus, Cinnamomi Cortex Spissus, Puerariae Radix, Zingiberis Rhizoma, Zizyphi Fructus |
| 55. Gamissangwhatang3 (加味双和汤3) | Paeoniae Radix, Rehmanniae Radix Preparata, Astragal Radix, Angelicae Gigantis Radix, Cnidii Rhizoma, Cinnamomi Cortex Spissus, Lycii Fructus, Polygonati falcati Rhizoma, Liriopis Tuber, Schizandrae Fructus, Glycyrrhizae Radix, Zingiberis Rhizoma, Zizyphi Fructus |
| 56. GamiikkiChongmyungtang (加味益气聪明汤) | Hoelen cum Pini Radix, Testudinis Plastrum Colla, Aurantii nobilis Pericarpium, Crataegi Fructus, Pachyma hoelen rumphius, Atractylodis Rhizoma, Atractylodis Rhizoma Alba, Astragal Radix, Hordei Fructus Germinatus, Alismatis Rhizoma, Gastrodiae Rhizoma, Zingiberis siccatum Rhizoma, Cervi cornu Colla, Phellodendri Cortex, Zingiberis Rhizoma |
| 57. Gamidanggwisusan (加味当归鬚散) | Angelicae Gigantis Radix, Cyperi Rhizoma, Linderae Radix, Caesalpiniae Lignum, Paeoniae Radix rubra, Cinnamomi Ramulus |
| 58. Biwibobang (脾胃补方) | Atractylodis Rhizoma, Aurantii nobilis Pericarpium, Machili Cortex, Dinelliae Tuber, Hoelen, Agastachis Herba, Gingseng Radix, Paeoniae Radix, Angelicae Gigantis Radix, Cnidii Rhizoma, Cinnamomi Cortex Spissus, Astragal Radix, Longanae Arillus, Glycyrrhizae Radix, Amomi Cardamomi Fructus, Amomi Semen, Zingiberis Rhizoma, Zizyphi Fructus, Amomi Tsao-ko Fructus |
| 59. Ssangwhatang1 (双和汤1) | Paeoniae Radix, Rehmanniae Radix Preparata, Astragal Radix, Angelicae Gigantis Radix, Cnidii Rhizoma, Cassiae Cortex, Glycyrrhizae Radix, Zingiberis Rhizoma, Zizyphi Fructus |
| 60. Daeyoungjeon2 (大营煎2) | Rehmanniae Radix Preparata, Angelicae Gigantis Radix, Lycii Fructus, Eucommiae Cortex, Achyranthis Radix, Glycyrrhizae Radix |
| 61. Ssangwhatanggagam1 (双和汤加减1) | Paeoniae Radix, Rehmanniae Radix Preparata, Astragal Radix, Angelicae Gigantis Radix, Cnidii Rhizoma, Cassiae Cortex, Polygonati falcati Rhizoma, Glycyrrhizae Radix |
| 62. Gwakhyangjeonggisan2 (藿香正气散2) | Agastachis Herba, Perillae Herba, Angelicae dahuricae Radix, Arecae Pericarpium, Machili Cortex, Atractylodis Rhizoma Alba, Aurantii nobilis Pericarpium, Dinelliae Tuber, Platycadi Radix, Glycyrrhizae Radix Praeparata, Zingiberis Rhizoma, Zizyphi Fructus |
| 63. Sibjeondaebotang2 (十全大补汤加味方2) | Cyperi Rhizoma, Angelicae Gigantis Radix, Cnidii Rhizoma, Paeoniae Radix, Rehmanniae Radix Preparata, Astragal Radix, Gingseng Radix, Atractylodis Rhizoma Alba, Pachyma hoelen rumphius, Glycyrrhizae Radix, Cinnamomi Cortex Spissus, Amomi Semen, Aurantii nobilis Pericarpium |
| 64. Heosusan (回首散) | Chaenomelis Fructus, Erythrinae Cortex, Aurantii nobilis Pericarpium, Linderae Radix, Osterici Radix, Araliae Cordatae Radix |
| 65. Yukmijihwangtanggagam (六味地黄汤加减) | Rehmanniae Radix Preparata, Dioscoreae Rhizoma, Corni Fructus, Liriopis Tuber, Moutau Cortex, Pachyma hoelen rumphius, Alismatis Rhizoma, Schizandrae Fructus |
| 66. Paljintanggamibang (八珍汤加味方) | Cyperi Rhizoma, Angelicae Gigantis Radix, Cnidii Rhizoma, Paeoniae Radix, Rehmanniae Radix Preparata, Gingseng Radix, Atractylodis Rhizoma Alba, Pachyma hoelen rumphius, Glycyrrhizae Radix, Amomi Semen |
| 67. Palmultanggagam2 (八物汤加减2) | Gingseng Radix, Astragal Radix, Angelicae Gigantis Radix, Hoelen, Cnidii Rhizoma, Atractylodis Rhizoma Alba |
| 68. Galgeunhaegitang2 (葛根解肌汤2) | Puerariae Radix, Bupleuri Radix, Scutellariae Radix, Paeoniae Radix, Osterici Radix, Platycadi Radix |
| 69. Gamigojineumja (加味固眞飮子) | Rehmanniae Radix Preparata, Gingseng Radix, Angelicae Gigantis Radix, Astragal Radix, Lycii Fructus, Phellodendri Cortex |
| 70. Dokhwaljihwangtanggagambang (独活地黄汤加减方) | Rehmanniae Radix Preparata, Corni Fructus, Hoelen, Alismatis Rhizoma, Moutau Cortex, Ledebouriellae Radix, Araliae Cordatae Radix, Testudinis Plastrum Colla, Amydae Carapax Colla |
| 71. ChungyeulsaseupTang (淸热泻湿汤) | Atractylodis Rhizoma, Phellodendri Cortex, Perillae Herba, Paeoniae Radix rubra, Chaenomelis Fructus, Alismatis Rhizoma |
| 72. Gamibojungikkitang4 (加味補中益氣湯4) | Astragal Radix, Gingseng Radix, Atractylodis Rhizoma Alba, Glycyrrhizae Radix, Angelicae Gigantis Radix, Aurantii nobilis Pericarpium |
| 73. Samchulkunbitang (蔘朮健脾汤) | Gingseng Radix, Atractylodis Rhizoma Alba, Pachyma hoelen rumphius, Machili Cortex, Aurantii nobilis Pericarpium, Corni Fructus, Paeoniae Radix, Crataegi Fructus, Ponciri Fructus, Pachyma hoelen rumphius, Amomi Semen, Massa Medicata Fermentata, Hordei Fructus Germinatus, Glycyrrhizae Radix |
| 74. Gagampyeongwisa (加减平胃散) | Atractylodis Rhizoma, Aurantii nobilis Pericarpium, Machili Cortex, Glycyrrhizae Radix, Zingiberis Rhizoma, Zizyphi Fructus, Ponciri Fructus, Coptidis Rhizoma |
| 75. Mahwangjeongcheontang (麻黄定喘汤) | Ephedrae Herba, Armeniacae Semen, Scutellariae Radix, Raphani Semen, Mori Cortex Radicis, Platycadi Radix, Liriopis Tuber, Perillae Semen, Schizandrae Fructus, Trichosanthes kirilowii, Perilla Frutescens |
| 76. Danggwisusantang (当归鬚散汤) | Angelicae Gigantis Radix, Paeoniae Radix rubra, Linderae Radix, Cyperi Rhizoma, Caesalpiniae Lignum, Carthami Flos, Persicae Semen, Cinnamomi Cortex Spissus, Glycyrrhizae Radix |
| 77. Ssangwhatang2 (双和汤2) | Paeoniae Radix, Astragal Radix, Angelicae Gigantis Radix, Cnidii Rhizoma, Rehmanniae Radix Preparata, Cassiae Cortex, Zizyphi Fructus, Zingiberis Rhizoma, Glycyrrhizae Radix |
| 78. Gagamjakgangtang (加减芍姜汤) | Paeoniae Radix, Glycyrrhizae Radix, Corydalis Tuber, Trogopterorum Faeces, Amomi Semen, Aurantii nobilis Pericarpium, Trtici Immaturi Semen, Zizyphi Fructus, Amomi Tsao-ko Fructus |
| 79. Gamgitang (感气汤) | Rehmanniae Radix Preparata, Raphani Semen, Astragal Radix, Atractylodis Rhizoma Alba, Dinelliae Tuber, Pachyma hoelen rumphius |
| 80. Jaeumbogantang (滋阴补肝汤) | Paeoniae Radix, Glycyrrhizae Radix, Scrophulariae Radix, Codonopsis Radix, Angelicae Gigantis Radix, Atractylodis Rhizoma Alba, Dioscoreae Rhizoma, Hordei Fructus Germinatus, Lycii Fructus, Glycyrrhizae Radix, Tribuli Fructus, Albizziae Cortex, Aurantii Immaturi Pericarpium, Artemisiae capillaris Herba, Curcumae Tuber, Galli Stomachichum Corium |
| 81. Sibjeondaebotanggamibang (十全大补汤加味方) | Rehmanniae Radix Preparata, Astragal Radix, Gingseng Radix, Angelicae Gigantis Radix, Cnidii Rhizoma, Atractylodis Rhizoma Alba, Hoelen, Glycyrrhizae Radix, Paeoniae Radix, Cinnamomi Cortex Spissus, Zingiberis Rhizoma, Zizyphi Fructus |
| 82. Gamiligitang (加味利气汤) | Aurantii nobilis Pericarpium, Aurantii Immaturi Pericarpium, Lithospermi Radix, Scutellariae Radix, Puerariae Radix, Perillae Herba, Cyperi Rhizoma, Paeoniae Radix, Amomi Tsao-ko Fructus, Crataegi Fructus, Trichosanthes kirilowii, Cannabis Semen, Zingiberis siccatum Rhizoma |
| 83. Whangkumgabanhansaenggangtang (黄芩加半夏生姜汤) | Dinelliae Tuber, Scutellariae Radix, Zizyphi Fructus, Zingiberis Rhizoma, Paeoniae Radix, Glycyrrhizae Radix |
| 84. Sibchiljeondaebotang (十七全大补汤) | Cervi cornu, Rehmanniae Radix Preparata, Astragal Radix, Paeoniae Radix, Angelicae Gigantis Radix, Cnidii Rhizoma |
| 85. Soakwiryongtang (加味归茸汤) | Rehmanniae Radix Preparata, Dioscoreae Rhizoma, Corni Fructus, Lycii Fructus, Moutau Cortex, Alismatis Rhizoma |
| 86. Sokyungwhalhyultang (疎经活血汤) | Atractylodis Rhizoma, Achyranthis Radix, Chaenomelis Fructus, Osterici Radix, Clematidis Radix, Coicis Semen |
| 87. Yikgeebohyultang (益气补血汤) | Astragal Radix, Angelicae Gigantis Radix, Longanae Arillus, Cyperi Rhizoma, Atractylodis Rhizoma Alba, Glycyrrhizae Radix |
| 88. Ojeoksangami (五积散加味) | Eucommiae Cortex, Phlomidis Radix, Carthami Flos, Zingiberis siccatum Rhizoma, Saussureae Radix, Persicae Semen |
| 89. Samwhatanggami (三和汤加味) | Perillae Herba, Cnidii Rhizoma, Linderae Radix, Corydalis Tuber, Gardeniae Fructus, Salviae Radix |
| ***From Dispensary of Oriental medicine (31 formulae)*** | |
| 90. Yongdamsagantang (龍胆泻肝汤) | Genianae Scabrae Radix, Alismatis Rhizoma, Plantayinis Semen, Rhemanniae Radix, Gardeniae Fructus, Glycyrrhizae Radix, Bupleuri Radix, Akebiae Caulis, Hoelen, Angelicae Gigantis Radix, Scutellariae Radix |
| 91. Soshihotang (小柴胡汤) | Bupleuri Radix, Dinelliae Tuber, Zingiberis Rhizoma, Scutellariae Radix, Gingseng Radix, Glycyrrhizae Radix |
| 92. Daeganghwaltang (大羌活汤) | Osterici Radix, Araliae Cordatae Radix, Stephaniae Tetrandrae Radix, Atractylodis Rhizoma Alba, Hoelen, Glycyrrhizae Radix, Cimicifugae Rhizoma, Atractylodis Rhizoma, Clematidis Radix, Angelicae Gigantis Radix, Alismatis Rhizoma |
| 93. Gamiondamtang1 (加味温胆汤1) | Aurantii nobilis Pericarpium, Hoelen, Ponciri Fructus, Scutellariae Radix, Liriopis Tuber, Dinelliae Tuber, Glycyrrhizae Radix, Bambusae Calulis in Taeniam, Coptidis Rhizoma, Phragmitis Rhizoma |
| 94. Gamipaedoksan (加味败毒散) | Osterici Radix, Anthrisci sylvestris Radix, Platycadi Radix, Hoelen, Glycyrrhizae Radix, Rhei Rhizoma, Araliae Cordatae Radix, Bupleuri Radix, Gingseng Radix, Aurantii Fructus, Cnidii Rhizoma, Atractylodis Rhizoma |
| 95. BanhabackchulchunmaTang (半夏白朮天麻汤) | Dinelliae Tuber, Aurantii nobilis Pericarpium, Hoelen, Atractylodis Rhizoma Alba, Gastrodiae Rhizoma, Glycyrrhizae Radix |
| 96. Ligigeopoongsan (理气祛风散) | Osterici Radix, Aurantii Fructus, Aurantii nobilis Pericarpium, Platycadi Radix, Dinelliae Tuber, Cnidii Rhizoma, Nepetae Herba, Paeoniae Radix, Araliae Cordatae Radix, Aurantii Immaturi Pericarpium, Linderae Radix, Arisaematis Rhizoma, Gastrodiae Rhizoma, Angelicae dahuricae Radix, Ledebouriellae Radix, Glycyrrhizae Radix |
| 97. Socheongryongtang (小靑龙汤) | Ephedrae Herba, Paeoniae Radix, Zingiberis siccatum Rhizoma, Glycyrrhizae Radix, Dinelliae Tuber, Asiasari Radix, Cinnamomi Ramulus, Schizandrae Fructus |
| 98. Daekumeumja (对金飮子) | Aurantii nobilis Pericarpium, Machili Cortex, Atractylodis Rhizoma, Glycyrrhizae Radix |
| 99. Wolbitang (越婢汤) | Ephedrae Herba, Gypsum, Zingiberis Rhizoma, Glycyrrhizae Radix, Zizyphi Fructus |
| 100. Baenongsanguptang (排脓散及汤) | Platycadi Radix, Glycyrrhizae Radix, Zizyphi Fructus, Paeoniae Radix, Zingiberis Rhizoma, Ponciri Fructus |
| 101. Sogeonjungtang (小建中汤) | Cinnamomi Ramulus, Paeoniae Radix, Zingiberis Rhizoma, Glycyrrhizae Radix, Zizyphi Fructus, Sacchrum Granorum |
| 102. Pyungwisan (平胃散) | Atractylodis Rhizoma, Machili Cortex, Aurantii nobilis Pericarpium, Glycyrrhizae Radix |
| 103. Saenghyeoryunbueum (生血润肤飮) | Asparagi Radix, Rehmanniae Radix Preparata, Angelicae Gigantis Radix, Scutellariae Radix, Persicae Semen, Carthami Flos, Rhemanniae Radix, Liriopis Tuber, Astragal Radix, Coicis Semen, Cimicifugae Rhizoma |
| 104. Ojeoksan3 (五积散3) | Atractylodis Rhizoma, Aurantii nobilis Pericarpium, Platycadi Radix, Angelicae Gigantis Radix, Paeoniae Radix, Angelicae dahuricae Radix, Dinelliae Tuber, Glycyrrhizae Radix, Ephedrae Herba, Machili Cortex, Aurantii Fructus, Zingiberis siccatum Rhizoma, Pachyma hoelen rumphius, Cnidii Rhizoma, Cassiae Cortex |
| 105. Jaeumkunbitang (滋阴建脾汤) | Atractylodis Rhizoma Alba, Dinelliae Tuber, Angelicae Gigantis Radix, Rhemanniae Radix, Hoelen cum Pini Radix, Polygalali Radix, Glycyrrhizae Radix, Aurantii nobilis Pericarpium, Pachyma hoelen rumphius, Paeoniae Radix, Gingseng Radix, Liriopis Tuber, Cnidii Rhizoma |
| 106. GwichulpajingTang (归朮破癥汤) | Cyperi Rhizoma, Zedoariae Rhizoma, Paeoniae Radix, Aurantii Immaturi Pericarpium, Carthami Flos, Cinnamomi Cortex Spissus, Scirpi Rhizoma, Paeoniae Radix rubra, Angelicae Gigantis Radix, Linderae Radix, Caesalpiniae Lignum |
| 107. Jaeumgangwhatang (滋阴降火汤) | Paeoniae Radix, Rehmanniae Radix Preparata, Atractylodis Rhizoma Alba, Rhemanniae Radix, Anemarrhenae Rhizoma, Glycyrrhizae Radix, Angelicae Gigantis Radix, Asparagi Radix, Liriopis Tuber, Aurantii nobilis Pericarpium, Phellodendri Cortex |
| 108. Sagunjatang (四君子汤) | Gingseng Radix, Glycyrrhizae Radix, Hoelen, Atractylodis Rhizoma Alba |
| 109. Nokyongdaebotang (鹿茸大补汤) | Boschniakiae Herba, Paeoniae Radix, Aconiti Tuber, Cinnamomi Cortex Spissus, Dendrobii Herba, Cervi Parvum cornu, Angelicae Gigantis Radix, Rehmanniae Radix Preparata, Eucommiae Cortex, Atractylodis Rhizoma Alba, Gingseng Radix, Dinelliae Tuber, Schizandrae Fructus, Astragal Radix, Pachyma hoelen rumphius, Glycyrrhizae Radix |
| 110. Samultang1 (四物汤1) | Angelicae Gigantis Radix, Cnidii Rhizoma, Paeoniae Radix, Rehmanniae Radix Preparata |
| 111. Guibitang1 (归脾汤1) | Angelicae Gigantis Radix, Zizyphi Spinosi Semen, Gingseng Radix, Atractylodis Rhizoma Alba, Saussureae Radix, Longanae Arillus, Polygalali Radix, Astragal Radix, Hoelen cum Pini Radix, Glycyrrhizae Radix |
| 112. Ssangwhatang3 (双和汤3) | Paeoniae Radix, Rehmanniae Radix Preparata, Astragal Radix, Angelicae Gigantis Radix, Cnidii Rhizoma, Cinnamomi Ramulus, Glycyrrhizae Radix |
| 113. Samryungbeakchulsan (參苓白朮散) | Gingseng Radix, Pachyma hoelen rumphius, Glycyrrhizae Radix, Nelumbinis Semen, Amomi Semen, Atractylodis Rhizoma Alba, Dioscoreae Rhizoma, Coicis Semen, Platycadi Radix, Dolichoris Semen |
| 114. Sibjeondaebotang3 (十全大补汤3) | Gingseng Radix, Cnidii Rhizoma, Hoelen, Astragal Radix, Paeoniae Radix, Cinnamomi Cortex Spissus, Rehmanniae Radix, Glycyrrhizae Radix, Angelicae Gigantis Radix, Atractylodis Rhizoma Alba |
| 115. Bunsimgieum (分心气飮) | Perillae Herba, Dinelliae Tuber, Aurantii Immaturi Pericarpium, Akebiae Caulis, Arecae Pericarpium, Saussureae Radix, Mori Cortex Radicis, Hoelen, Zedoariae Rhizoma, Platycadi Radix, Arecae Semen, Liriopis Tuber, Cyperi Rhizoma, Glycyrrhizae Radix, Ponciri Fructus, Aurantii nobilis Pericarpium, Cinnamomi Ramulus, Agastachis Herba |
| 116. Oyaksunkisan (乌药顺气散) | Ephedrae Herba, Linderae Radix, Angelicae dahuricae Radix, Aurantii Fructus, Zingiberis siccatum Rhizoma, Aurantii nobilis Pericarpium, Cnidii Rhizoma, Bombycis Corpus, Platycadi Radix, Glycyrrhizae Radix |
| 117. Chungganhaewooltang (淸肝解郁汤) | Gingseng Radix, Fritillariae Bulbus, Rehmanniae Radix Preparata, Atractylodis Rhizoma Alba, Bupleuri Radix, Aurantii nobilis Pericarpium, Moutau Cortex, Hoelen, Gardeniae Fructus, Paeoniae Radix, Angelicae Gigantis Radix, Cnidii Rhizoma, Glycyrrhizae Radix |
| 118. Yeonryeonggobondan (延龄固本丹) | Asparagi Radix, Rhemanniae Radix, Dioscoreae Rhizoma, Eucommiae Cortex, Schizandrae Fructus, Corni Fructus, Gingseng Radix, Thujae Semen, Acori Graminei Rhizoma, Alismatis Rhizoma, Rubi Fructus, Cuscutae Semen, Liriopis Tuber, Rehmanniae Radix Preparata, Achyranthis Radix, Morindae Radix, Lycii Fructus, Pachyma hoelen rumphius, Saussureae Radix, Zanthoxyli Fructus, Polygalali Radix, Boschniakiae Herba, Plantayinis Semen, Lycii Cortex Radicis |
| 119. Chungseoikgitang (淸暑益气汤) | Atractylodis Rhizoma, Cimicifugae Rhizoma, Atractylodis Rhizoma Alba, Massa Medicata Fermentata, Phellodendri Cortex, Puerariae Radix, Liriopis Tuber, Astragal Radix, Gingseng Radix, Aurantii nobilis Pericarpium, Alismatis Rhizoma, Angelicae Gigantis Radix, Aurantii Immaturi Pericarpium, Glycyrrhizae Radix |
| 120. Chungsangbangpoongtang (淸上防风汤) | Ledebouriellae Radix, Forsythiae Fructus, Coptidis Rhizoma, Scutellariae Radix, Angelicae dahuricae Radix, Aurantii Fructus, Nepetae Herba, Gardeniae Fructus, Menthae Herba, Cnidii Rhizoma, Platycadi Radix, Glycyrrhizae Radix |
| ***From herbal medicine shop (35 formulae)*** | |
| 121. Ssanghwatang2 (双和汤2) | Paeoniae Radix, Cnidii Rhizoma, Angelicae Gigantis Radix, Astragal Radix, Cinnamomi Cortex Spissus |
| 122. Palmultang (八物汤) | Atractylodis Rhizoma Alba, Pachyma hoelen rumphius, Gingseng Radix, Glycyrrhizae Radix, Rehmanniae Radix Preparata |
| 123. Sibjeondaebotang4 (十全大补汤4) | Rehmanniae Radix Preparata, Astragal Radix, Angelicae Gigantis Radix, Paeoniae Radix, Cnidii Rhizoma |
| 124. Yukmiwongagam (六味元加减) | Glycyrrhizae Radix, Dioscoreae Rhizoma, Corni Fructus, Moutau Cortex, Alismatis Rhizoma |
| 125. Daebotang (大补汤) | Angelicae Gigantis Radix, Gastrodiae Rhizoma, Paeoniae Radix, Cnidii Rhizoma, Glycyrrhizae Radix |
| 126. Ssangwhatang4 (双和汤4) | Cnidii Rhizoma, Paeoniae Radix, Rehmanniae Radix Preparata, Cassiae Cortex, Astragal Radix |
| 127. Sibjeondaebotang5 (十全大补汤5) | Rehmanniae Radix Preparata, Angelicae Gigantis Radix, Cnidii Rhizoma, Paeoniae Radix, Astragal Radix |
| 128. Yukmijihwangtang1 (六味地黄汤1) | Rehmanniae Radix Preparata, Corni Fructus, Lycii Fructus, Rubi Fructus, Alismatis Rhizoma |
| 129. Sibjeondaebotang6 (十全大补汤6) | Angelicae Gigantis Radix, Cnidii Rhizoma, Paeoniae Radix, Atractylodis Rhizoma Alba, Glycyrrhizae Radix |
| 130. Guibitang2 (归脾汤2) | Longanae Arillus, Angelicae Gigantis Radix, Astragal Radix, Polygalali Radix, Hoelen cum Pini Radix |
| 131. Yukmissangwhatang (六味双和汤) | Paeoniae Radix, Rehmanniae Radix Preparata, Angelicae Gigantis Radix, Cnidii Rhizoma, Astragal Radix |
| 132. Ssangwhatang5 (双和汤5) | Glycyrrhizae Radix, Paeoniae Radix, Astragal Radix, Rehmanniae Radix Preparata, Cnidii Rhizoma |
| 133. Gamidaebotang1 (加味大补汤1) | Angelicae Gigantis Radix, Cnidii Rhizoma, Eucommiae Cortex, Achyranthis Radix, Chaenomelis Fructus |
| 134. Gamidaebotang2 (加味大补汤2) | Coicis Semen, Angelicae Gigantis Radix, Rhei Rhizoma, Atractylodis Rhizoma, Glycyrrhizae Radix |
| 135. Gamidaebotang3 (加味大补汤3) | Eucommiae Cortex, Acanthopanacis Cortex, Aurantii nobilis Pericarpium, Glycyrrhizae Radix, Hoelen |
| 136. Mangeumtang (万金汤) | Prunus nakii, Arecae Semen, Angelicae Gigantis Radix, Rehmanniae Radix Preparata, Boschniakiae Herba |
| 137. Sibjeondaebotang7 (十全大补汤7) | Angelicae Gigantis Radix, Cnidii Rhizoma, Paeoniae Radix, Rehmanniae Radix Preparata, Astragal Radix |
| 138. Palmultanggagam3 (八物汤加减3) | Pachyma hoelen rumphius, Aurantii nobilis Pericarpium, Puerariae Radix, Glycyrrhizae Radix, Crataegi Fructus |
| 139. Jueryeongtanggagam (猪苓汤加减) | Akebiae Caulis, Plantayinis Semen, Genianae Scabrae Radix, Cervi cornu, Glycyrrhizae Radix |
| 140. Bojungikkitanggagam3 (补中益气汤加减3) | Astragal Radix, Angelicae Gigantis Radix, Schizandrae Fructus, Achyranthis Radix, Dioscoreae Rhizoma |
| 141. Yukmijihwangtang2 (六味地黄汤2) | Dioscoreae Rhizoma, Corni Fructus, Alismatis Rhizoma, Hoelen, Rehmanniae Radix Preparata |
| 142. Ssangpaetang (双败汤) | Angelicae Gigantis Radix, Cnidii Rhizoma, Paeoniae Radix, Rehmanniae Radix Preparata, Astragal Radix |
| 143. Sibjeondaebotang8 (十全大补汤8) | Astragal Radix, Angelicae Gigantis Radix, Cnidii Rhizoma, Paeoniae Radix, Cassiae Cortex |
| 144. Yukmijihwangtang3 (六味地黄汤3) | Dioscoreae Rhizoma, Corni Fructus, Moutau Cortex, Alismatis Rhizoma, Hoelen |
| 145. Gamidaebotang4 (加味大补汤4) | Angelicae Gigantis Radix, Cnidii Rhizoma, Paeoniae Radix, Rehmanniae Radix Preparata, Astragal Radix |
| 146. Soyosan (逍遥散) | Atractylodis Rhizoma Alba, Paeoniae Radix, Hoelen, Bupleuri Radix, Angelicae Gigantis Radix |
| 147. Sojagangkitang (苏子降气汤) | Dinelliae Tuber, Perillae Semen, Aurantii nobilis Pericarpium, Angelicae Gigantis Radix, Machili Cortex |
| 148. Yukmijihwangtang4 (六味地黄汤4) | Rehmanniae Radix Preparata, Corni Fructus, Dioscoreae Rhizoma, Hoelen, Alismatis Rhizoma |
| 149. Hwangnyeonhaedoktang (黄连解毒汤) | Scutellariae Radix, Coptidis Rhizoma, Phellodendri Cortex, Gardeniae Fructus |
| 150. Dokhwalgisaengtang (独活寄生汤) | Araliae Cordatae Radix, Angelicae Gigantis Radix, Paeoniae Radix, Loranthi Ramulus, Rehmanniae Radix Preparata |
| 151. Samultang2 (四物汤2) | Angelicae Gigantis Radix, Cnidii Rhizoma, Paeoniae Radix, Rehmanniae Radix Preparata |
| 152. Bojungikkitanggagam4 (补中益气汤加减4) | Zizyphi Spinosi Semen, Longanae Arillus, Angelicae Gigantis Radix, Astragal Radix, Atractylodis Rhizoma Alba |
| 153. Gamiyangwitang (加味养胃汤) | Longanae Arillus, Astragal Radix, Lophatheri Herba, Cyperi Rhizoma, Crataegi Fructus |
| 154. Gamiondamtang2 (加味温胆汤2) | Longanae Arillus, Zizyphi Spinosi Semen, Astragal Radix, Dioscoreae Rhizoma, Aurantii nobilis Pericarpium |
| 155. Ssangwhatanggagam2 (双和汤加减2) | Rehmanniae Radix Preparata, Paeoniae Radix, Astragal Radix, Cnidii Rhizoma, Cassiae Cortex |

| **Supplementary Table 2**  **Pesticides analyzed in herbal decoctions** | | | |
| --- | --- | --- | --- |
| Methods | Pesticide | Classification | Molecular Formula |
| GC/NPD  7 kinds | Cyprodinil  Iprobenfos  Napropamide  Tebuconazole  Tebufenpyrad  Triadimenol  Triazophos | Fungicide  Acaricide  Insecticide  Fungicide  Fungicide  Insecticide  Fungicide | C13H11Cl2NO2  C18H24ClN3O  C9H21O2PS3  C10H13Cl2FN2O2S2  C14H16ClN3O2  C12H16N3O3PS  C15H15ClF3N3O |
| GC/μECD  26 kinds | p,p'-DDD  p,p'-DDE  o,p'-DDT  p,p'-DDT  Bifenthrin  Chlorfenapyr  Chlorothalonil  Cyhalothrin  Cypermethrin  Dieldrin  α-Endosulfan  β-Endosulfan  Endosulfan Sulfate  Fenarimol  Fenpropathrin  Hexaconazole  Isoprothiolane  Kresoxim-methyl  Methoxychlor  Pendimethalin  Procymidone  Tetradifon  Thifluzamide  Tolylfluanid  Triadimefon  Triflumizole | Insecticide  Insecticide  Insecticide  Fungicide  Insecticide  Fungicide  Fungicide  Insecticide  Fungicide  Insecticide  Insecticide  Insecticide  Insecticide  Insecticide  Insecticide  Insecticide  Insecticide  Insecticide  Fungicide  Fungicide  Fungicide  Fungicide  Insecticide  Insecticide  Fungicide  Herbicide | C9H11Cl3NO3PS  C23H19ClF3NO3  C22H19Cl2NO3  C14H15N3  C6H6Cl6  C9H8Cl3NO2S  C10H6N2OS2  C15H11BrClF3N2O  C8Cl4N2  C14H9Cl5  C14H9Cl5  C14H9Cl5  C14H9Cl5  C9H6Cl6O3S  C9H6Cl6O3S  C9H6Cl6O4S  C12H8Cl6O  C8H19O2PS2  C17H12Cl2N2O  C12H6F2N2O2  C13H21O3PS  C18H19NO4  C16H15Cl3O2  C7H3Cl5S  C15H17ClN4  C17H21NO2 |

| **Supplementary Table 3.**  **Recovery for 33 pesticides at three concentration levels** | | | | |
| --- | --- | --- | --- | --- |
| Pesticides | Mean recovery ± RSD(%)^1)^ | | | Average |
|  | 0.5mg/kg | 1.0mg/kg | 2.0mg/kg |  |
| o,p'-DDT  p,p'-DDD  p,p'-DDE  p,p'-DDT  Bifenthrin  Chlorfenapyr  Chlorothalonil  Cyhalothrin  Cypermethrin  Dieldrin  α-Endosulfan  β-Endosulfan  Endosulfan Sulfate  Fenarimol  Fenpropathrin  Hexaconazole  Isoprothiolane  Kresoxim-methyl  Methoxychlor  Pendimethalin  Procymidone  Tetradifon  Thifluzamide  Tolyfluanide  Triadimefon  Acetamiprid  Cyprodinil  Iprobenfos  Napropamid  Tebuconazole  Tebufenpyrad  Triadimenol  Triazophos | 108.5 ± 1.2  108.3 ± 1.9  59.7 ± 4.9  99.2 ± 2.5  95.3 ± 2.2  86.0 ± 0.8  65.6 ± 6.8  86.1 ± 0.9  99.0 ± 3.3  77.6 ± 5.7  30.2 ± 1.6  80.4 ± 1.6  99.0 ± 1.4  80.7 ± 1.4  95.8 ± 2.1  93.2 ± 4.3  80.7 ± 1.0  90.7 ± 2.0  76.2 ± 2.8  62.0 ± 1.3  102.1 ± 1.7  91.3 ± 2.1  99.6 ± 2.4  60.4 ± 0.9  80.4 ± 0.8  96.1 ± 1.6  103.9 ± 8.0  83.0 ± 3.7  68.4 ± 3.4  77.0 ± 0.2  96.4± 6.0  84.1± 3.8  69.7 ± 7.0 | 81.6 ± 1.6  85.6 ± 1.1  46.0 ± 1.8  89.7 ± 3.4  104.9 ± 0.4  88.6 ± 0.9  74.4 ± 2.4  85.5 ± 2.4  108.6 ± 1.6  85.7 ± 2.0  30.5 ± 1.2  81.6 ± 1.3  101.6 ± 1.4  86.5 ± 1.9  96.6 ± 2.0  84.9 ± 1.0  89.1 ± 1.9  99.3 ± 0.7  82.5 ± 0.8  52.9 ± 0.2  97.3 ± 2.0  102.1± 0.6  90.5 ± 2.1  66.1 ± 1.2  77.6 ± 1.7  113.0 ± 3.1  98.3 ± 3.3  86.1± 1.1  71.2± 3.1  71.2 ± 2.5  93.7 ± 5.3  82.5 ± 4.7  74.7 ± 5.3 | 98.0 ± 0.7  97.3 ± 4.6  52.1 ± 2.1  98.9 ± 1.7  106.1 ± 0.6  91.0 ± 1.4  73.0 ± 2.1  89.0 ± 1.7  115.3 ± 0.7  77.4 ± 5.6  32.5 ± 1.0  90.2 ± 1.1  105.5 ± 0.8  89.7 ± 0.9  100.9 ± 0.5  84.8 ± 1.5  103.1 ± 1.2  100.5 ± 0.3  86.9 ± 0.2  56.8 ± 2.3  97.4 ± 1.6  108.4 ± 0.8  99.3 ± 0.6  69.1 ± 1.3  87.0 ± 1.4  107.3 ± 7.1  91.3 ± 5.0  96.7 ± 1.2  61.4 ± 1.5  78.0 ± 9.2  86.7 ± 3.1  70.1 ± 8.3  74.9 ± 3.5 | 96.0 ± 14.1  97.0 ± 11.7  52.6 ± 13.0  96.0 ± 5.6  102.1 ± 5.8  88.5 ± 2.8  71.0 ± 6.6  86.9 ± 2.2  107.6 ± 7.6  80.2 ± 5.9  31.1 ± 4.1  84.1 ± 6.4  102.1 ± 3.2  85.6 ± 5.3  97.8 ± 2.8  87.7 ± 5.5  90.9 ± 12.4  96.8 ± 5.5  81.9 ± 6.5  57.2 ± 8.0  98.9 ± 2.8  100.6 ± 8.6  96.5 ± 5.4  65.2 ± 6.8  81.7 ± 5.9  105.5 ± 8.1  97.8 ± 6.5  88.6 ± 8.1  67.0 ± 7.5  75.4 ± 4.9  92.3 ± 5.4  78.9 ± 9.7  73.1 ± 4.1 |
| 1) RSD(%) = relative standard deviation ; (standard deviation /mean)x100 | | | | |
